# Characterization of pancreatic cancer with ultra-low tumor mutational burden

**DOI:** 10.1038/s41598-023-31579-8

**Published:** 2023-03-16

**Authors:** Taisuke Imamura, Ryo Ashida, Keiichi Ohshima, Katsuhiko Uesaka, Teiichi Sugiura, Katsuhisa Ohgi, Mihoko Yamada, Shimpei Otsuka, Keiichi Hatakeyama, Takeshi Nagashima, Takashi Sugino, Kenichi Urakami, Yasuto Akiyama, Ken Yamaguchi

**Affiliations:** 1 Division of Hepato-Biliary-Pancreatic Surgery, Shizuoka Cancer Center, 1007 Shimonagakubo, Sunto-Nagaizumi, Shizuoka, 4118777 Japan; 2 Medical Genetics Division, Shizuoka Cancer Center Research Institute, Shizuoka, Japan; 3 Cancer Multiomics Division, Shizuoka Cancer Center Research Institute, Shizuoka, Japan; 4 Cancer Diagnostics Research Division, Shizuoka Cancer Center Research Institute, Shizuoka, Japan; 5 SRL, Inc., Tokyo, Japan; 6 Division of Pathology, Shizuoka Cancer Center, Shizuoka, Japan; 7 Immunotherapy Division, Shizuoka Cancer Center Research Institute, Shizuoka, Japan; 8 Shizuoka Cancer Center Hospital and Research Institute, Shizuoka, Japan

**Keywords:** Pancreatic cancer, Prognostic markers

## Abstract

In pancreatic cancer (PC), Tumor mutation burden (TMB) has been reported to be lower than in other cancers, with its clinical significance remaining unclear. We analyzed the dataset of whole-exome sequencing and gene expression profiling of 93 resected PC cases. The median TMB was 0.24. The TMB was classified as High (≥ 5.0), Low (< 5.0, ≥ 1.0), or Ultra-low (< 1.0). Nineteen samples (20%) were classified as TMB-low, and 74 (80%) were classified as TMB-ultra-low; no samples were TMB-high. TMB-ultra-low PC had significantly fewer borderline resectable lesions (*P* = 0.028) and fewer adenosquamous carcinomas (*P* = 0.003) than TBM-low PC. Furthermore, the TMB-ultra-low PC showed significantly lower detection rates of driver mutations and copy number variations. Microsatellite instability was not significantly correlated with the TMB status. The TMB-ultra-low PC had a significantly better prognosis than TBM-low PC (*P* = 0.023). A multivariate analysis identified TMB-ultra-low PC as an independent favorable prognostic factor (hazard ratio, 2.11; *P* = 0.019). A gene expression analysis showed that TMB-ultra-low PC was associated with reduced *TP53* inactivation (*P* = 0.003) and reduced chromosomal instability (*P* = 0.001) compared to TBM-low PC. TMB-ultra-low PC had specific gene expression signatures and a better prognosis than TMB-low PC.

## Introduction

The tumor mutation burden (TMB) is an emerging feature of cancers that was first highlighted by large-scale mutational analyses using next-generation sequencing^1^. The TMB ranges from > 100 mut/Mb (melanoma, lung cancer) to as low as 0.1 mut/Mb (pediatric cancer) depending on the cancer type, and even within the same cancer type^2^. Not only driver mutations but even passenger mutations cause amino acid substitutions, resulting in antigen presentation of novel peptides that elicit an anti-tumor immune response^3,4^. In addition, it has been shown that immunocytolytic activity is associated with the accumulation of mutations in many solid tumors^5^. Based on these findings, high-TMB tumors are highly immunogenic, and immune checkpoint inhibitors (ICIs) are expected to be effective in the treatment of such tumors.

Clinical trials of ICI therapy for various types of solid tumors have revealed that a high TMB may be potential biomarkers for predicting the clinical response and prognosis^6–10^. A high TMB is accepted as a positive biomarker for antitumor effects and favorable prognosis of patients with MSI-H cancer^11–13^. In many types of cancers, however, TMB-high tumor are limited, and a majority of tumors are determined to be TMB-low^1,14^. However, the features of TMB-low tumors, which comprise the majority of solid tumors, are still poorly understood. A previous pan-cancer study revealed that PC belongs to the cancer type category with the lowest TMB^15^. For this reason, reports on the relationship between the TMB and clinical outcomes in PC are limited^16–18^.

Numerous studies have examined the optimal TMB cut-off values for determining the survival in cancer patients^19–22^. Recently, our pan-cancer study identified categories of cancer patients enrolled in Project HOPE in terms of the TMB: ultra-low (TMB-ultra-low: < 1 mutation per megabase), low (TMB-low: 1 to < 5 mutations per megabase), and high (TMB-high: ≥ 5 mutations per megabase); we then characterized the unique gene signatures of these categories^23^. The present results are a part of the single center study called “High-tech Omics-based Patient Evaluation” or “Project HOPE” conducted at the Shizuoka Cancer Center from 2014, which aims to establish the Japanese version of The Cancer Genome Atlas (JCGA). We previously reported the overall results of a multi-omics analysis of a 93-case pancreatic cancer cohort in the JCGA dataset, including whole-exome sequencing (WES), oncogene panel sequencing, fusion gene panel sequencing, and gene expression profiling (GEP) analyses^24^. In the present study, we focused on TMB and investigated the relationship between TMB and the clinicopathological and molecular features of PC. This analysis allowed us to characterize PCs with ultra-low TMB.

## Results

The clinicopathological characteristics are summarized in Table 1. For resectability classification, 88% of cases were R, and BR accounted for 12% of all cases. NAT was performed in all BR cases and was not performed in any R cases. Postoperative adjuvant therapy was performed in 70% of cases. This cohort included adenosquamous carcinoma (11%). The median follow-up period was 26.4 months. The 3- and 5- year overall survival (OS) rates were 39.8% and 22.2%, respectively.

**Table 1:** Clinical correlates according to the TMB status.

Variable	All	TMB		*P*-value
		Ultra-low (< 1.0)	Low (< 5.0, ≥ 1.0)	
	N = 93	N = 74	N = 19	
Patient characteristics				
Age (years)	71 (64–77)	71 (64–77)	73 (64–78)	0.853
Sex male				
Male	55 (59%)	46 (62%)	9 (47%)	0.242
Female	38 (41%)	28 (38%)	10 (53%)	
CA19-9 level (units/ml)	124 (32–577)	121 (31–596)	144 (31–507)	0.943
Peri-operative management				
Resectability status				
R	82 (88%)	68 (92%)	14 (74%)	**0.028**
BR	11 (12%)	6 (8%)	5 (26%)	
Neoadjuvant therapy				
Performed	11 (12%)	8 (11%)	3 (16%)	0.549
Adjuvant therapy				
Performed	65 (70%)	53 (72%)	12 (63%)	0.478
Pathological findings				
Location				
Ph	59 (63%)	46 (62%)	13 (68%)	0.613
Pbt	34 (37%)	28 (38%)	6 (32%)	
Histological type				
Adenocarcinoma	83 (89%)	70 (95%)	13 (68%)	**0.003**
Adenosquamous	10 (11%)	4 (5%)	6 (32%)	
Primary tumor status				
T1	2 (2%)	2 (3%)	0 (0%)	0.368
T2	63 (68%)	52 (70%)	11 (58%)	
T3	28 (31%)	20 (27%)	8 (42%)	
Nodal status				
N0	23 (25%)	18 (24%)	5 (26%)	0.977
N1	41 (44%)	33 (45%)	8 (42%)	
N2	29 (31%)	23 (31%)	6 (32%)	
Metastasis				
M0	89 (96%)	70 (95%)	19 (100%)	0.300
M1	4 (4%)	4 (5%)	0 (0%)	
Resection status				
R0	89 (96%)	70 (95%)	19 (100%)	0.300
R1	4 (4%)	4 (5%)	0 (0%)	

*BR* borderline resectable, *R* resectable, *Ph* pancreatic head, *Pbt* pancreatic body and tail, *TMB* tumor mutation burden. Significant values are in bold.

The molecular correlates are summarized in Table 2. The median estimated tumor content was 0.25 with a quartile range of 0.19–0.36. Driver mutations were identified in 61 cases (66%). The most frequent mutation was the *KRAS* mutation, found in 54 cases (58%), and MSI-high inferred from WES was found in only 3 cases (3%).

**Table 2:** Molecular correlates according to the TMB status.

Variable	All	TMB		*P*-value
		Ultra-low (< 1.0)	Low (< 5.0, ≥ 1.0)	
	N = 93	N = 74	N = 19	
Molecular correlates				
Estimated tumor content	0.25 (0.19–0.36)	0.24 (0.17–0.30)	0.34 (0.30–0.42)	**0.001**
Driver mutation (tier 1 or 2) identified	61 (66%)	42 (57%)	19 (100%)	**< 0.001**
Number of driver mutation identified	1 (0–2)	1 (0–2)	2 (1–3)	**< 0.001**
MSI-high	3 (3%)	2 (3%)	1 (5%)	0.573
CNV size (gain)	12.8 (0.0–103.3)	6.1 (0.0–39.6)	24.0 (3.4–66.0)	**< 0.001**
CNV size (loss)	24.0 (3.4–66.0)	19.6 (3.4–44.2)	65.6 (23.4–79.6)	**< 0.001**
* KRAS* mutation	54 (58%)	36 (49%)	18 (95%)	**< 0.001**
* TP53* mutation	36 (39%)	22 (30%)	14 (74%)	**0.001**
* CDKN2A* mutation	7 (8%)	3 (4%)	4 (21%)	**0.012**
* SMAD4* mutation	7 (8%)	5 (7%)	2 (11%)	0.593

*TMB* tumor mutation burden, *MSI* microsatellite instability, *CNV* copy number variants. Significant values are in bold.

### The comparison of the TMB between WES and CCP

We previously reported that the TMB according to WES is lower than that according to CCP, especially in samples with a low tumor cellularity (< 0.25)^25^. In addition, a study involving low-cellularity PC samples highlighted the importance of deep sequencing of low-purity samples^26^. We therefore compared the TMB calculated from WES with that calculated from deep sequencing results using CCP to examine which was more appropriate for exploring the characteristics of TMB-ultra-low PC.

**Figure 1:**
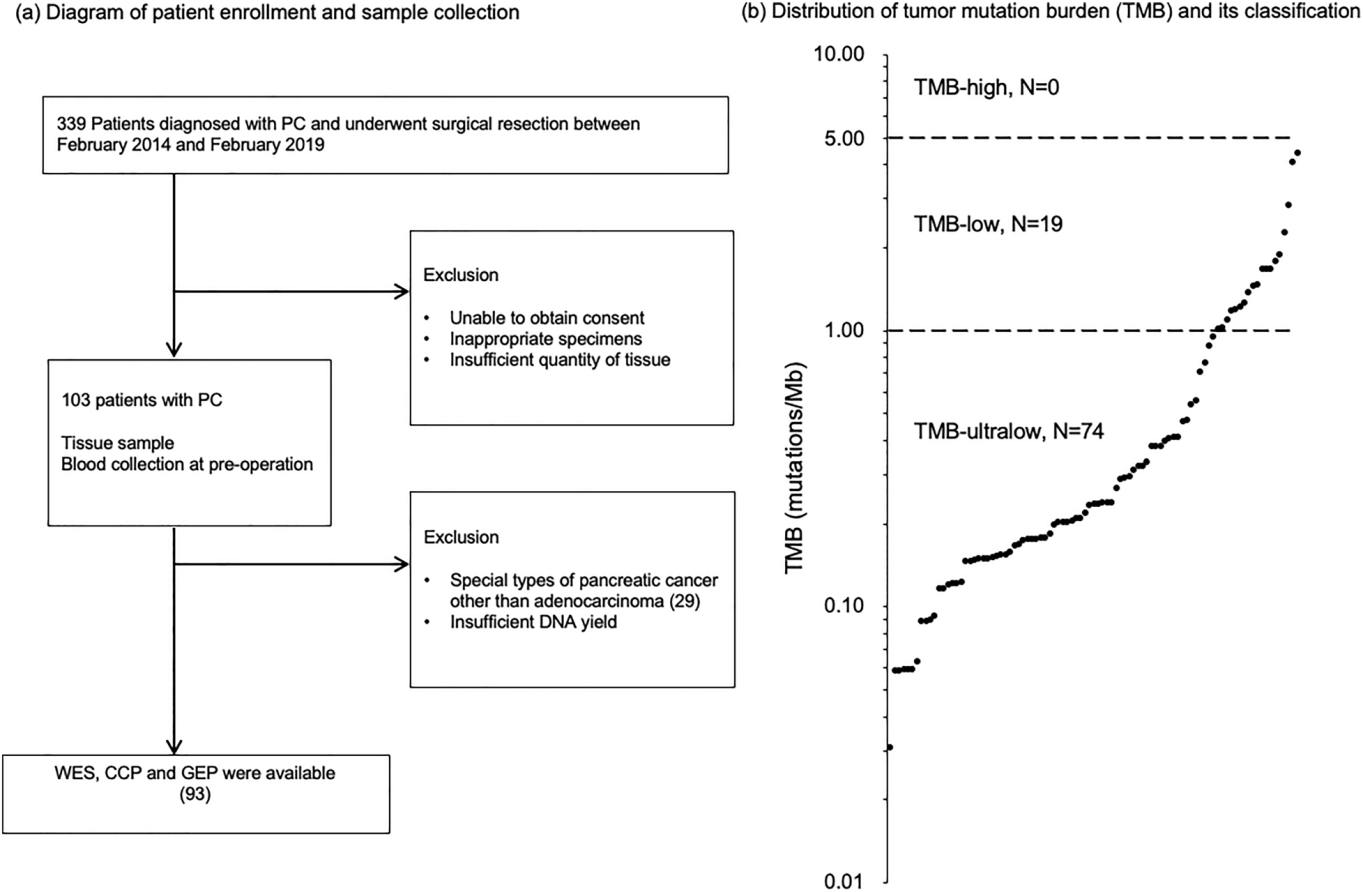
(**a**) Flow diagram of patient enrollment and sample collection. From January 2014 to March 2019, a total of 103 cases of PC were analyzed in Project HOPE. Tumor tissue samples were collected from resected specimens by pathologists, and all tumor tissues were pathologically diagnosed as PC. Ninety-three resected PC cases with available data for WES, CCP, and GEP were enrolled in this study. (**b**) Distribution of the TMB and its classification. The median TMB was 0.238, with an interquartile range of 0.15–0.64. No samples (0.0%) had a TMB ≥ 5.00 mutations/Mb (TMB-high). Nineteen samples (20%) had 1 to < 5 mutations/Mb, defined as TMB-low, and 74 tumors had < 1.0 mutation/Mb, defined as TMB ultra-low (80%).

The median TMB from WES was 0.24, with a quartile range of 0.15–0.64. The median TMB from CCP was 3.36 with a quartile range of 1.71–5.80. Scatter plots of the TMB from WES vs. the TMB from CCP are shown in Supplementary Fig. 1a. The values calculated using the 2 platforms showed intermediate correlation (*ρ* = 0.557). A correlation analysis of tumor cellularity with the TMB from WES revealed only a weak correlation (*ρ* = 0.31, Supplementary Fig. 1b), while there was little correlation between the tumor cellularity and the TMB from CCP (*ρ* = 0.17, Supplementary Fig. 1c). These results indicate that the impact of a low tumor content on the TMB from WES in PC is limited. Since there were no cases classified as TMB < 1, the definition of TMB-ultra-low, when using the TMB from CCP, we proceeded the analysis using the WES-based TMB. The distribution of TMB from WES is shown in Fig. 1b. No samples (0.0%) had TMB ≥ 5.00 mutations/Mb (TMB-high). The TMB status was annotated as TMB-low or TMB-ultra-low according to our previous report^23^. Nineteen samples (20%) had 1 to < 5 mutations/Mb, defined as TMB-low, and 74 had < 1.0 mutation/Mb, defined as TMB ultra-low (80%).

### Somatic mutations in TMB-ultra-low PC

**Figure 2:**
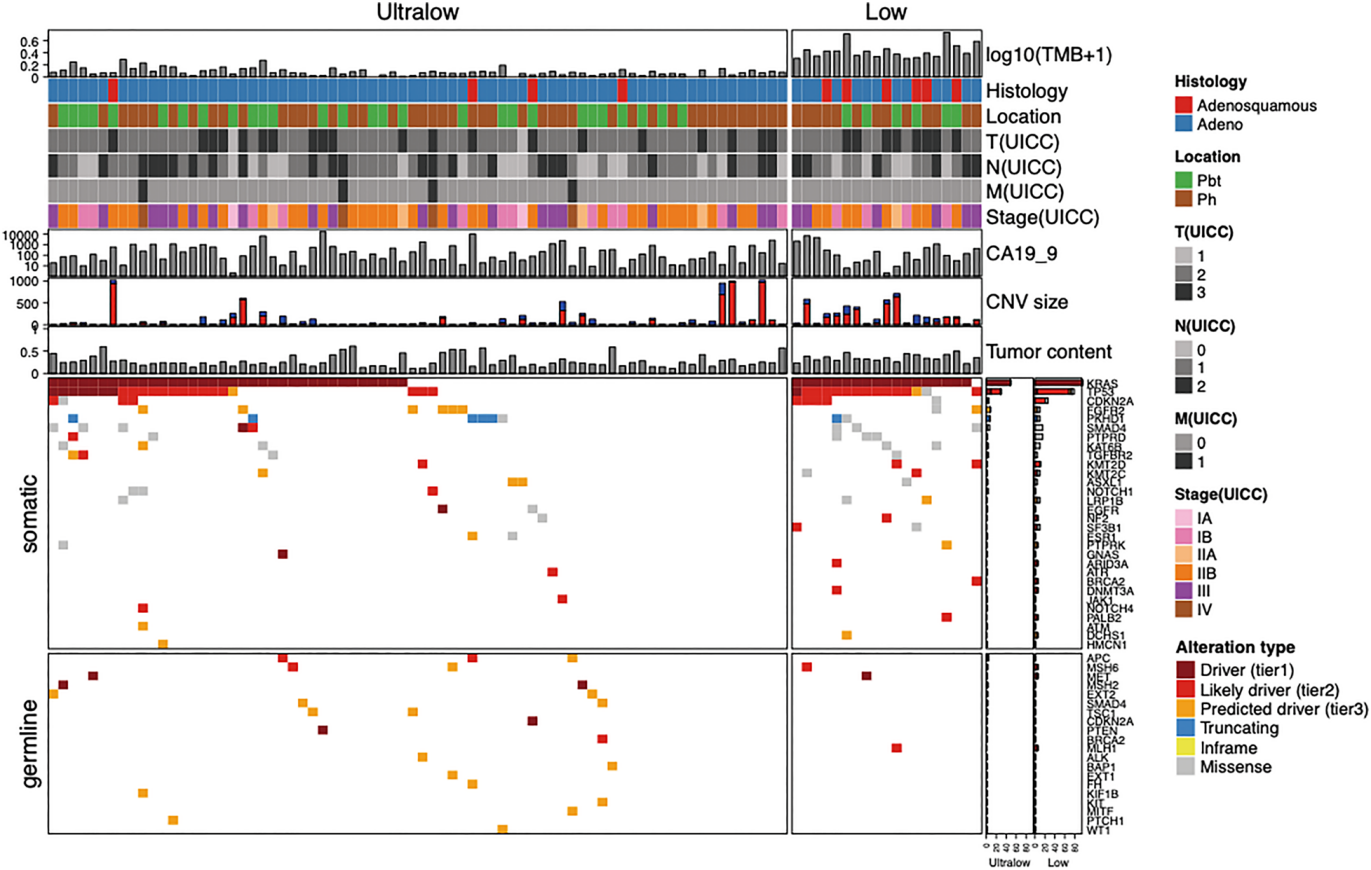
Landscape of genomic alterations according to the TMB classification in pancreatic cancer. The bar graph on the right shows the mutation frequency of each mutated gene. Green represents substitution/Indel mutations, red represents gene amplification mutations, blue represents gene homozygous deletion mutations, yellow represents fusion/rearrangement mutations, and purple represents truncation mutations.

We previously reported the landscape of mutations in PC in the series^24^. In the present study, we focused on TMB and analyzed the characteristics of mutations among subgroups classified according to the TMB. Figure 2 shows the landscape of genomic alterations in PC based on the TMB classification. A summary of the comparison of the molecular correlation according to the TMB classification is shown in Table 2. The detection rate of driver-mutated samples was investigated. The driver mutations were annotated as Tier 1 or 2 according to a previous report^27^. The frequency of driver mutations was decreased in TMB-ultra-low tumors compared with that in TMB-low tumors (*P* < 0.001). To evaluate the correlation between tumor cellularity and the detection of somatic alterations, estimated tumor cellularity was compared between the two groups. The tumor cellularity was higher in TMB-low tumors than in TMB-ultra-low tumors (median 0.34 vs. 0.24, *P* = 0.001). Somatic mutations were identified at a significantly higher frequency in TMB-low tumors than in TMB-ultra-low tumors in several genes, such as *KRAS* (*P* < 0.001), *TP53* (*P* = 0.001), and *CDKN2A* (*P* = 0.030). Furthermore, we also checked known fusion genes, but no fusion gene was detected in this pancreatic cancer study.

### CNV and TP53 inactivation in TMB ultra-low PC

**Figure 3:**
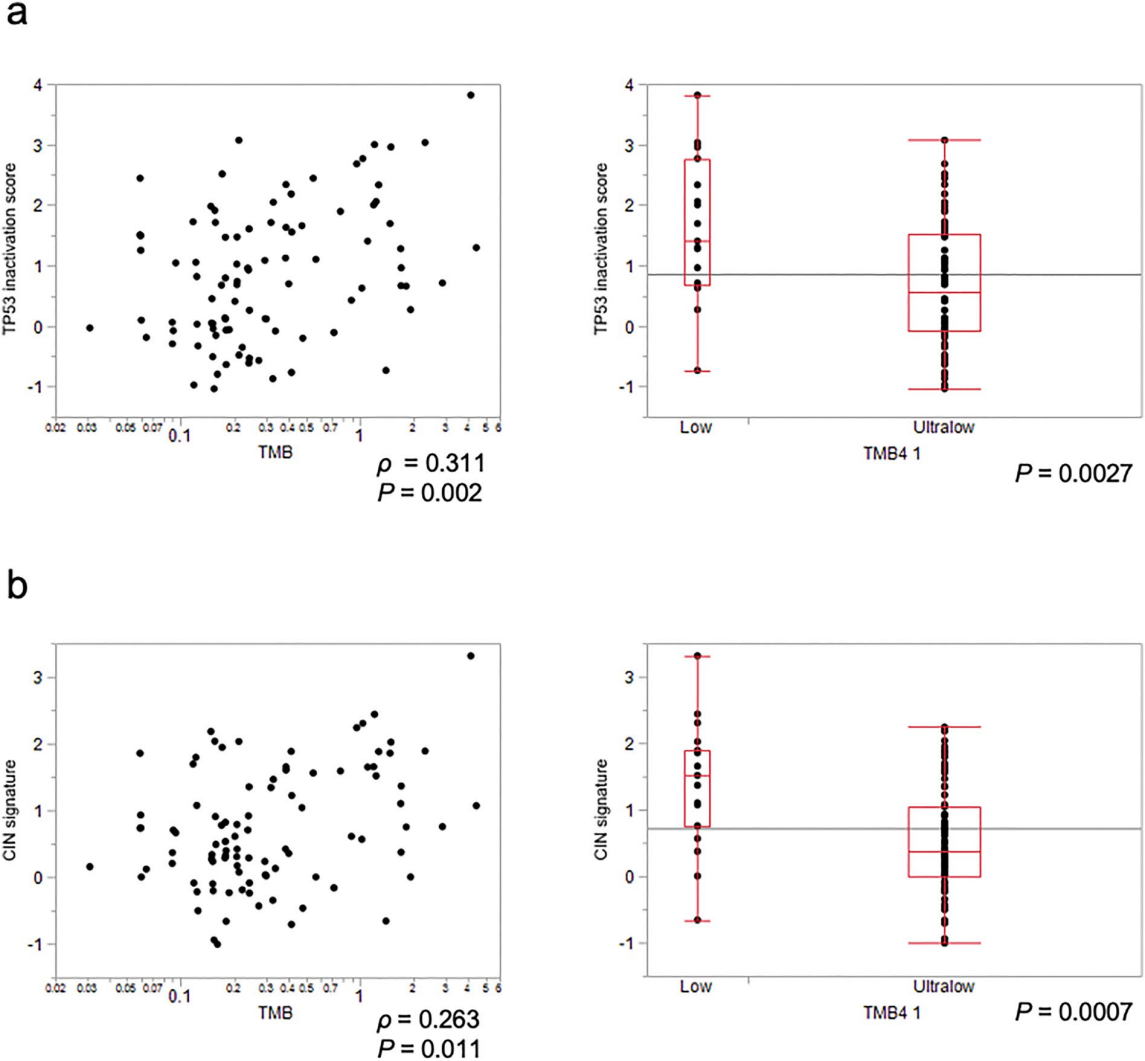
Relationship between the gene expression signatures and TMB. (**a**) The left figure shows the correlation between the TMB and TP53 inactivation score, and the right figure shows the comparison of the TP53 inactivation score after the classification of the TMB. The TP53 inactivation score was significantly decreased in TMB-ultra-low tumors. (**b**) The left figure shows the correlation between the TMB and CIN signature score, and the right figure shows the comparison of the CIN signature score after the classification of the TMB. The CIN signature score was significantly decreased in TMB-ultra-low tumors.

TMB-low tumors harbored more driver or cancer-related mutations than TMB-ultra-low tumors. To detect other alterations superseding driver mutations, we performed a CNV analysis (Fig. 3). Both the CNV size for gain (*P* < 0.001) and the CNV size for loss (*P* = 0.019) were extremely low in TMB-ultra-low tumors, implying that these TMB-ultra-low PC were less prone to somatic chromosome alterations and that the accumulation of CNV was lower in TMB-ultra-low tumors than in others. A defective *TP53* function is known to contribute to CIN progress. Based on a previous expression analysis^28^, we calculated the TP53 inactivation score. However, the TMB correlated only slightly with the TP53 inactivation score (*ρ* = 0.31, *P* = 0.002), which was significantly reduced in TMB-ultra-low tumors. This finding is consistent with that in a previous report (Fig. 3a). Furthermore, the TMB was also slightly correlated with the CIN signature^29^ (*ρ* = 0.26, *P* = 0.011), and the CIN signature score was significantly decreased in TMB-ultra-low tumors (*P* = 0.001 Fig. 3b).

### Response prediction of ICI in TMB-ultralow tumors

MSI-high inferred from WES was found in only 3 cases (3%) in our cohort. There was no significant difference in the frequency of MSI-high between TMB-ultra-low PC and TMB-low PC (*P* = 0.573). A combination of TMB and GEP related to T cell activation can improve the prediction of responses to ICI^30–32^. To explore a candidate responder to ICI therapy and further characterize TMB-ultralow tumors, the distribution of the T cell-inflamed GEP signature was investigated. Our analysis revealed that there was no significant correlation between the TMB and T cell-inflamed gene expression profiling signatures (*ρ* = 0.025, *P* = 0.871). In addition, our analysis identified no significant difference in the T cell-inflamed GEP signature between TMB-ultra-low PC and TMB-low PC (*P* = 0.407, Supplementary Fig. 2).

### Clinicopathological features and outcomes in TMB-ultra-low PC

**Figure 4:**
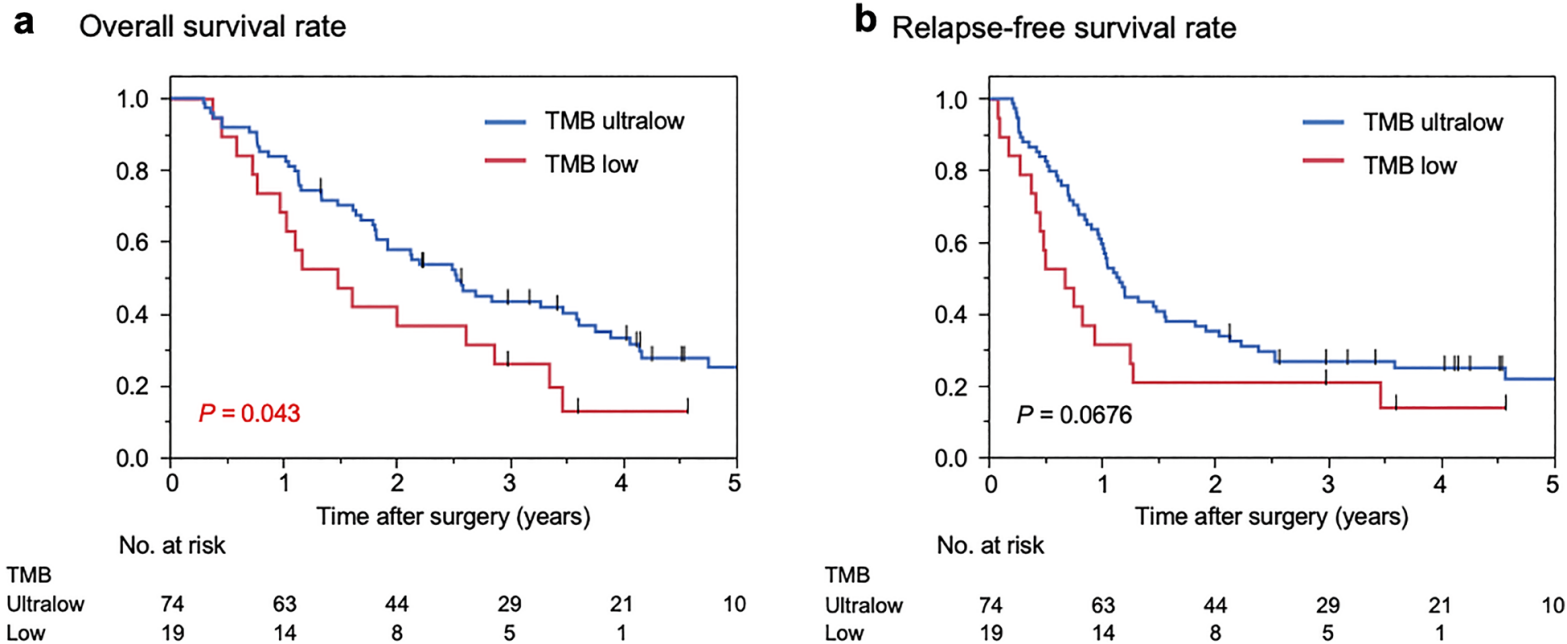
A prognostic analysis according to the TMB classification. (**a**) In the TMB-ultra-low group, the OS was significantly better than in the TMB-low group (median, 39.2 vs. 19.2 months; *P* = 0.023). (**b**) TMB-low PC tended to have a poorer recurrence-free survival (RFS) than TMB-ultralow, although the difference in the RFS did not reach significance (median, 8.1 vs. 13.9 months; *P* = 0.068).

The clinicopathological factors according to the TMB definitions are shown in Table 1. The comparison of TMB-ultra-low PC with TMB-low PC revealed that TMB-ultra-low PC had significantly fewer borderline resectable lesions (*P* = 0.028) and significantly fewer adenosquamous carcinomas (*P* = 0.003) than TBM-low PC. In the TMB-ultra-low group, the OS was significantly better than in the TMB-low group (median, 39.2 vs. 19.2 months; *P* = 0.023, Fig. 4a). TMB-low PC tended to have a poorer recurrence-free survival (RFS) than TMB-ultralow, although the difference in the RFS just barely does not reached significance (median, 8.1 vs. 13.9 months; *P* = 0.068, Fig. 4b). A Cox proportional hazards analysis for the OS (Table 2) identified TMB classification as an independent prognostic factor (hazard ratio [HR], 2.57; *P* = 0.001) as well as adjuvant treatment (HR, 2.20; *P* = 0.007) and lymph node metastasis (HR, 3.40; *P* = 0.001). Estimated tumor content and driver mutations, which were associated with the TMB, were not associated with the OS, suggesting that these factors are not confounded by the TMB being an independent prognostic factor for the OS.

To investigate the impact of the TMB on the patterns of recurrence, the correlations between TMB classification and the initial recurrence site and time after resection were assessed (Table 3). Although there were no significant differences in the frequency, distant metastasis (*P* = 0.036) and early recurrence within 6 months after surgery (*P* = 0.006) were significantly less frequently observed in the TMB-ultra-low group than in others (Table 4).

**Table 3:** Univariate and multivariate analyses for the overall survival after surgery.

Variable	Univariate		Multivariate	
	*P*-value^a^	HR	95% CI	*P*-value^b^
Patient characteristics				
Age, years				
≥ 70	0.416			
< 70				
Sex male				
Male	0.779			
Female				
CA19-9 level, units/ml				
≥ 124	**0.045**	1.13	0.68–1.88	0.6321
< 124				
**Peri-operative management**				
Resectability status				
BR	0.221			
R				
Neoadjuvant treatment				
Not performed	0.982			
Performed				
Adjuvant treatment				
Not performed	**0.003**	1.50	0.88–2.51	0.1360
Performed				
Pathological findings				
Location				
Ph	**0.013**	1.15	0.66–2.07	0.6292
Pbt				
Histological type				
Adenocarcinoma	0.958			
Adenosquamous carcinoma				
Tumor size, mm				
≥ 40	0.815			
< 40				
Lymph node metastasis				
Positive	**< 0.001**	4.18	1.95–10.06	**< 0.001**
Negative				
Distant metastasis				
Positive	0.851			
Negative				
Surgical margin				
Positive	0.929			
Negative				
Molecular correlates				
TMB				
Low (< 5.0, ≥ 1.0)	**0.018**	2.11	1.14–3.73	**0.0192**
Ultra-low (< 1.0)				
Estimated tumor content				
≥ 0.25	0.609			
< 0.25				
Driver mutations				
KRAS mutant	0.060			
KRAS wild type				
TP53 mutant	0.339			
TP53 wild type				
CDKN2A mutant	0.175			
CDKN2A wild type				
SMAD4 mutant	0.761			
SMAD4 wild type				

*HR* hazard ratio, *CI* confidence interval, *BR* borderline resectable, *R* resectable, *Ph* pancreatic head, *Pbt* pancreatic body and tail, *TMB* tumor mutation burden. Significant values are in bold.

^a^Kaplan-Meier method. Significance was determined by the log-rank test. ^b^Multivariate survival analysis was performed using Cox's proportional hazard model.

**Table 4:** Sites of recurrence according to TMB in pancreatic cancer patients who underwent surgical resection.

Variable	All	TMB		*P*-value
		Ultra-low (< 1.0)	Low (< 5.0, ≥ 1.0)	
	N = 93	N = 74	N = 19	
Recurrence	71 (76%)	56 (76%)	15 (79%)	0.765
Site				
Locoregional	30 (32%)	26 (35%)	4 (21%)	0.242
Distant	49 (53%)	35 (47%)	14 (74%)	**0.036**
Peritoneal	13 (14%)	10 (14%)	3 (16%)	0.799
Timing				
Early phase (< 6 months)	21 (23%)	12 (16%)	9 (47%)	**0.006**

*BR* borderline resectable, *R* resectable, *Ph* pancreatic head, *Pbt* pancreatic body and tail, *TMB* tumor mutation burden. Significant values are in bold.

## Discussion

While a few studies have analyzed the characteristics of tumors with a high TMB in PC^18^, there are still no studies focusing on PC tumors with a low TMB. Therefore, we focused on tumors with a particularly low TMB among PC cases, defined as TMB-ultra-low PC and attempted to characterize these samples using WES and GEP. In the present study, the median TMB in PC tumors was found to be 0.24, which is very low compared to other cancers; there were no TMB-high (TMB ≥ 5.0) tumors, 20% were classified as TMB-low (< 5.0, ≥ 1.0), and 80% were classified as TMB-ultra-low (< 1.0). The TMB-ultra-low PC had a significantly better prognosis than TBM-low PC. A multivariate analysis identified TMB-ultra-low PC as an independent favorable prognostic factor.

Previous studies investigating TMB in resected PC^18^ reported that a TMB ≥ 10 mutations/Mb accounted for 7% of cases, 10 > TMB > 5 mutations/Mb accounted for 18%, and TMB ≤ 5 mutations/Mb accounted for 75%. This reported TMB is low compared to other cancers described thus far but is slightly higher than our present results, being generally consistent. In addition, those authors also revealed that the prognosis of patients could be stratified by the TMB value. However, their finding that tumors with a high TMB have a better prognosis and tumors with low TMB have a poorer prognosis than others differs from the trend in the present study, where TMB-ultra-low PC had a better prognosis than TMB-low PC. This discrepancy in the survival may be due to the cut-off value of the TMB. Previous studies placed cases with a TMB < 5, which accounts for a large portion of their PC cohort (75%), into a single category and did not focus on ultra-low-TMB cases (< 1.0) within that category or explore its characteristics. In the present study, we focused on ultra-low-TMB PC cases among PC, as this subset is representative of cancers with a particularly low TMB, and characterized the details for the first time.

Clinicopathologically, the TMB-ultra-low PC contained a higher rate of R-PC and lower rate of BR-PC. Furthermore, TMB-ultra-low PC included fewer cases of adenosquamous carcinoma, which is reported to have a poorer prognosis than adenocarcinoma^33^. These clinicopathologic factors may have contributed to the particularly favorable prognosis of TMB-ultra-low PC. Indeed, only in the adenocarcinoma subgroup, there was no significant difference in the prognosis between the TMB-low and TMB-ultralow groups (*P* = 0.308). These results suggest that adenosquamous cell carcinoma may contribute to the poor prognosis in the TMB-low group. The mutational analysis showed that TMB-ultra-low PC had a significantly lower frequency of driver mutations and a significantly lower number of driver mutations than TMB-low PC. Since driver mutations have been reported to be a poor prognostic factor in PC^34,35^, the possibility that differences in the frequency of driver mutations may be confounded by the results of the prognostic analysis should be considered. However, as shown in Table 3, in the present study, individual driver mutations were not significantly identified as prognostic factors in the prognostic factor analysis, although TMB was identified as an independent prognostic factor. We believe that this result supports TMB as a prognostic factor in resected PC and the favorable prognosis for TMB-ultra-low PC in contrast to the general recognition that low-TMB PC has a poor prognosis.

In our previous pan-cancer study^36^, the association of TMB status and immune parameters, such as the expression of specific immune response genes, specific immune cell populations, and TCR repertoire profile were investigated. The analysis of immune cell populations in tumors demonstrated that the frequencies of exhausted CD8^+^ T-cells, activated effector CD8^+^ T-cells, and natural killer cells were significantly lower in TMB-ultra-low tumors. The T-cell receptor repertoire numbers and diversity evenness score (DE50) were higher in the TMB-ultra-low tumors. One possible mechanism underlying the relatively good prognosis of the TMB-ultra-low PC might be that the TMB-ultra-low PC features a balance between immunosuppression and immunostimulation. We plan to further investigate the precise mechanism responsible for these observations in the future.

Deficiency of *TP53* activation has been recognized to be associated with enhanced CIN^28,37^. Our gene expression signature analysis showed that TMB-ultra-low PC was associated with decreased *TP53* inactivation and decreased CIN compared to TBM-low PC. Therefore, the TMB-ultra-low tumors may be less likely to accumulate somatic mutations and CNVs because TP53 activation is maintained. This means that TMB-ultra-low PC may also include tumors that have developed through other tumorigenic processes besides *TP53* inactivation. A recent pan-cancer analysis revealed that chromothripsis(-like) events often occur in tumors with few driver mutations^38^. Conversely, recent mutational analyses have shown that composite mutations led to tumorigenesis^39,40^. Although the involvement of these mechanisms is speculated, to further understand the details of TMB-ultra-low PC, the integration of our analysis and whole-genome sequencing findings, including those of our germline mutation analysis, should be explored in the future.

Thus far, immunotherapy using ICIs has not been successful implemented in PC, mostly due to the immunosuppressive tumor microenvironment and the relatively low expression and/or low quality of tumor-specific neoantigens^41,42^. The prediction of responses to ICIs using gene expression focuses on tumors harboring some mutations (≥ 10 mutations/Mb)^30–32^, although most solid tumors were categorized as TMB-low. Our analysis revealed that there was no notable correlation between the TMB and T cell-inflamed gene expression profiling signatures, indicating treatment responsiveness to ICIs (*P* = 0.407). Although a high expression of T-cell inflammatory signatures in a population might be expected to indicate candidate ICI responsiveness despite a low number of mutations, the existing evidence concerning the correlation between the TMB and neoantigen expression and the efficacy of ICIs in PC is rather weak^43,44^. Immunotherapy for PC with ICIs and their biomarkers should be explored further in the future.

PC is characterized by a marked desmoplastic reaction with dense fibrous stroma and low neoplastic cellularity^45^. This low neoplastic cellularity can confound the analysis of the mutational and gene expression characteristics in actual tumor cells. Our previous pan-cancer study excluded samples with low tumor cellularity (< 0.3) from the mutation analysis. In the present PC study, the median estimated tumor content was 0.25. Adopting the threshold of 0.3 would exclude a large portion of samples from the analysis. This low tumor content is one concern as a limitation. A study involving low-cellularity PC samples highlighted the importance of deep sequencing of low-purity samples^26^. We therefore compared the WES-calculated TMB with the TMB calculated from deep sequencing results using CCP to determine which was more appropriate for exploring the characteristics of TMB-ultra-low PC. The results indicated that the effect of a low tumor content on the TMB determined using WES was limited in PC. Notably, in the prognostic analysis, the TMB was identified as an independent prognostic factor, even though the tumor content was not identified as a prognostic factor even in univariate analysis. Based on these results, it is reasonable to assume that TMB-ultra-low PC has a better prognosis than TMB-low PC, although the tumor content is somewhat confounded by the TMB. Prior genome sequencing studies have focused on tumors with neoplastic cellularity > 40% 45 or purified tumor samples using laser capture microdissection techniques^46^. The low tumor content and lack of microdissection or other devices to increase the tumor content are limitations of this study. Nevertheless, further investigation of PC-specific testing methods and appropriate cutoff values may be warranted.

In conclusion, the present study characterized PC tumors with a particularly low TMB, defined as TMB-ultra-low PC in the JCGA dataset. Detection rates of driver mutations and CNV were decreased in TMB-ultra-low PC. TMB-ultra-low PC was associated with decreased TP53 inactivation and decreased CIN. Furthermore, among tumors with low TMB, which have been considered a poor prognosis group based on the results of previous studies, we identified TMB-ultra-low PC as a subgroup of tumors with a relatively good prognosis. Our analysis focusing on TMB-ultra-low PC can provide insight into PC with low numbers of somatic alterations.

## Methods

### Ethics statement

This project HOPE was designed according to the “Ethical Guidelines for Human Genome and Genetic Analysis Research,” revised in 2013^47^. Written consent was obtained from all patients participating in Project HOPE. The present study used data from Project HOPE and was approved by the Institutional Review Board of Shizuoka Cancer Center (approval no. 25-33). All experiments using clinical samples were performed in accordance with the approved Japanese ethical guidelines (human genome/gene analysis research, 2017, provided by Ministry of Health, Labor, and Welfare; https://www.mhlw.go.jp/stf/seisakunitsuite/bunya/hokabunya/kenkyujigyou/i-kenkyu/index.html).

### Patients and samples

From January 2014 to March 2019, a total of 103 cases of PC were analyzed in Project HOPE. Tumor tissue samples were collected from resected specimens by pathologists, and all tumor tissues were pathologically diagnosed as PC. Tumor-adjacent tissue specimens were used as the normal control for microarrays. In addition, to identify somatic and germline genomic alterations in patients, peripheral blood was collected as a pair control for excluding germline mutations for WES, comprehensive cancer panel (CCP) and fusion gene panel^48^ sequencing for tumor tissue. Ninety-three resected PC cases with available data in WES, CCP, and GEP were enrolled in this study (Fig. 1a).

### Data sets for the analysis of somatic alterations

The detailed experimental protocols and procedures used to analyze the sequence and microarray data have been previously described^27^. In brief, the exome library for WES was constructed using an Ion Torrent AmpliSeq RDY Exome Kit (Thermo Fisher Scientific). The exome library supplied 292 903 amplicons covering 57.7 Mb of the human genome, comprising 34.8 Mb of exonic sequences from 18 835 genes registered in RefSeq. These data were then submitted to the National Bioscience Database Center (NBDC) Human Database as ‘Controlled-Access Data’ (Research ID, hum0127.v1; https://humandbs.biosciencedbc.jp/en/).

For somatic mutations in CCP, variant calls were conducted for both normal and tumor specimens using the Torrent Variant Caller, and the difference between tumor and normal specimens was considered a somatic mutation. To detect cancer driver mutations with high sensitivity, the threshold of VAF for SNV detection was changed from 2 to 0.5%, and the threshold of VAF in mutations registered in COSMIC was changed from 1 to 0.1%.

Tumor cellularity was estimated by averaging two individual algorithms (FACETS and Sequenza) as described in a previous study^7^. Somatic genomic alterations contributing to tumorigenesis were extracted from multiple databases and then curated in-house as driver mutations^7^. Somatic CNVs were detected using saasCNV^26^. This method accounted for both the read-depth ratio and B allele frequency and achieved the best performance among six CNV detection tools^27^.

### Gene expression signature analyses

Purified total RNA for GEP was then amplified and fluorescently labeled using a One-Color Low Input Quick Amp Labeling Kit (Agilent Technologies). Cy3-labeled cRNAs were hybridized to a SurePrint G3 Human Gene Expression 8 × 60K v2 Microarray (Agilent Technologies). A signature analysis based on the gene expression was performed using the expression ratio of the tumor and the corresponding normal tissue (T/N). The expression signature/score was calculated from the average of genes in unique gene sets corresponding to each individual signature (TP53 inactivation^28^, chromosomal instability [CIN]^49^).

### Treatment strategy for PC

The resectability status was classified as resectable (R), borderline resectable (BR), and unresectable (UR) according to the NCCN guidelines^50^. During the study periods, the standard treatment for RPC at our institution was upfront surgical resection followed by postoperative adjuvant chemotherapy (AC) consisting of gemcitabine or S-1 for six months. AC mainly constituted S-1^51^. Neoadjuvant treatment (NAT) for BR-PC was introduced, and patients with BR-PC received S-1/RT, gemcitabine plus nab-paclitaxel (GnP), or FOLFIRINOX (leucovorin, 5-fluorouracil, irinotecan, and oxaliplatin). Patients with initially URPC underwent conversion surgery were not enrolled in the study. Surgical resection was performed as previously described^52^. Resected specimens were examined by pathologists and evaluated based on the 8th edition of the American Joint Committee on Cancer/Union for International Cancer Control (AJCC/UICC) staging system^53^.

### Statistical analyses

Continuous variables were expressed as the median with the interquartile range (IQR) and were compared using the Mann–Whitney *U* test. Categorical variables were compared using Fisher’s exact test. The survival was calculated using the Kaplan–Meier method, and the log-rank test was used to evaluate the statistical significance of the differences.

All statistical analyses were performed using the JMP software package (version 14.0 for Mac; SAS Institute Inc., Cary, NC, USA). *P* values of < 0.05 were considered to indicate statistical significance.

### Ethics approval and consent to participate

Clinical samples from patients were obtained after acquiring the consent of the patient in accordance with the protocol approved by the Ethics Boards of Shizuoka Cancer Center. This study was performed in accordance with the Declaration of Helsinki.

## Supplementary Information

Supplementary Figures.

## Data Availability

All data and materials generated during and/or analyzed during this study are available from the corresponding author on reasonable request. Data for this study are confidential patient information regulated by the IRB of the institution. The datasets generated and/or analyzed during the current study are available in the National Bioscience Database Center repository (accession no. hum0127; https://humandbs.biosciencedbc.jp/en/).
