# Supplementary material for: Characterization of pancreatic cancer with ultra-low tumor mutational burden
Source: Sci Rep. 2023 Mar 16;13:4359. doi: 10.1038/s41598-023-31579-8 (PMC10020557; doi:10.1038/s41598-023-31579-8)
Supplement: Supplementary file 1 — Supplementary Figures. [file 41598_2023_31579_MOESM1_ESM.docx]

**Supplementary Fig.1.** *The comparison of the TMB between WES and CCP*

1. Scatter plots of the TMB from WES vs. the TMB from CCP
2. A correlation analysis of tumor cellularity with the TMB from WES
3. A correlation analysis of tumor cellularity with the TMB from CCP

**Supplementary Fig.2.** *Response prediction of ICI in TMB-ultralow tumors*

1. Scatter plots of the TMB from WES vs. the TMB from CCP
2. A comparison of T cell-inflamed GEP signature between TMB-ultra-low PC and TMB-low PC
